# Stochastic Competition between Mechanistically Independent Slippage and Death Pathways Determines Cell Fate during Mitotic Arrest

**DOI:** 10.1371/journal.pone.0015724

**Published:** 2010-12-21

**Authors:** Hsiao-Chun Huang, Timothy J. Mitchison, Jue Shi

**Affiliations:** 1 Department of Systems Biology, Harvard Medical School, Boston, Massachusetts, United States of America; 2 Graduate Program in Systems Biology, Harvard Medical School, Boston, Massachusetts, United States of America; 3 Center for Quantitative Systems Biology and Department of Physics, Hong Kong Baptist University, Kowloon Tong, Hong Kong; Roswell Park Cancer Institute, United States of America

## Abstract

Variability in cell-to-cell behavior within clonal populations can be attributed to the inherent stochasticity of biochemical reactions. Most single-cell studies have examined variation in behavior due to randomness in gene transcription. Here we investigate the mechanism of cell fate choice and the origin of cell-to-cell variation during mitotic arrest, when transcription is silenced. Prolonged mitotic arrest is commonly observed in cells treated with anti-mitotic drugs. Cell fate during mitotic arrest is determined by two alternative pathways, one promoting cell death, the other promoting cyclin B1 degradation, which leads to mitotic slippage and survival. It has been unclear whether these pathways are mechanistically coupled or independent. In this study we experimentally uncoupled these two pathways using zVAD-fmk to block cell death or Cdc20 knockdown to block slippage. We then used time-lapse imaging to score the kinetics of single cells adopting the remaining fate. We also used kinetic simulation to test whether the behaviors of death versus slippage in cell populations where both pathways are active can be quantitatively recapitulated by a model that assumes stochastic competition between the pathways. Our data are well fit by a model where the two pathways are mechanistically independent, and cell fate is determined by a stochastic kinetic competition between them that results in cell-to-cell variation.

## Introduction

Individual cells choose between alternative states in many aspects of biology. In most cases that have been investigated, the decision is made by gene regulation. Single-cell studies on randomness in mRNA transcription show that stochastic gene expression plays a crucial role in bacterial competence, microbial survival and blood cell differentiation [1]–[3]. Here, we investigate how cells choose between alternative fates during mitotic arrest induced by anti-mitotic drugs, where transcription is silenced due to chromosome condensation, and decision-making must occur by purely post-transcriptional mechanisms. Understanding how cells chose between survival and death in response to a therapeutically important drug class may help improve cancer chemotherapy.

**Figure 1:**
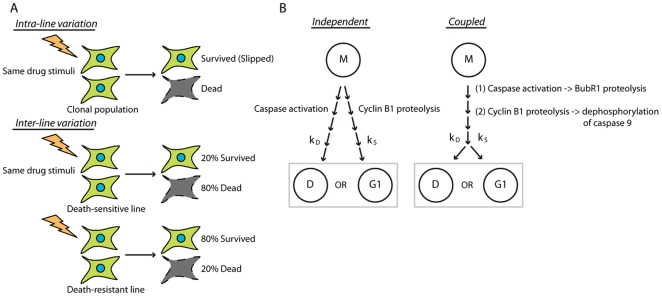
Kinetic models explaining intra- and inter-line variations. (A) Intra- and inter-line variations under the same drug stimuli. For intra-line variation, non-genetic variations in protein levels or post-translational modifications among individual cells results in different cell fates within a clonal population. For inter-line variation, the average behavior, or overall percentage of cell death/survival, is determined by genetic factors. (B) Models of kinetic competition between death and slippage. For independent pathway competition, mitotic state triggers two independent processes, caspase activation and cyclin B1 proteolysis, each leading to its corresponding state. For coupled pathway competition, mitotic states triggers one signal that is triggering the other, and the two coupled processes compete with their kinetic rates. M: mitosis; D: death; G1: post-slippage G1.

One common cancer chemo-therapeutic strategy is to perturb cell division with anti-mitotic drugs. Anti-mitotic drugs that target microtubules (Taxanes, Vinca Alkaloids) are mainstays of current chemotherapy, and new classes of anti-mitotics that specifically interfere with the assembly of bipolar mitotic spindles by inhibiting various mitotic kinases or kinesin motor proteins are under development [4]. The primary targets of these drug classes differ, but their cellular effects converge on disrupting mitotic spindle assembly, leading to chronic failure to satisfy the spindle assembly checkpoint (SAC). As a result, the cell cycle stalls in mitotic arrest that lasts many hours. Some fraction of dividing cancer cells are killed during or after this mitotic arrest, but typically not 100%. Incomplete cell killing could be a problem for therapy, and its mechanistic origin has been unclear. Recent single-cell studies have shed light on variability in the drug response. In all cell lines that have been studied, saturating drug concentrations causes long-lasting mitotic arrest in 100% of the population. However, subsequent behaviors vary profoundly. Individual cells may die during the mitotic arrest, or slip out of the arrest into a tetraploid G1 state, from which they may die, arrest in G1, or continue the cell cycle [5]–[12]. Variation in cell fate was observed both as a difference in individual cell behavior within a clonal population (intra-cell line variation) and also as a difference in average behavior between different cell lines (inter-cell line variation) (Fig. 1A). It prompts the question, what kind of internal calculation determines whether a given cell lives and slips or dies following mitotic arrest, and why do individual cells vary in the result of this calculation?

We know that cell fate during mitotic arrest is controlled by two alternative pathways, one that promotes slippage out of mitosis and cellular survival from mitotic stress, the other that promotes cell death [13]. Mitotic slippage occurs because cyclin B1 is slowly and progressively proteolyzed during mitotic arrest by the same APC/C proteosome pathway that promotes normal mitotic exit when the SAC is satisfied [14]. Eventually, cyclin B1 drops to a low enough level that the cell can no longer sustain the mitotic state, and it exits into a tetraploid G1 state. Compared to the slippage pathway, the pathway that triggers cell death in mitotic arrest is less clear. It culminates in mitochondrial outer membrane permeabilization (MOMP) and activation of capsases 9, 3 and 7. The events that precede MOMP are not well understood; we and others hypothesize that some progressive biochemical or organizational change accumulates over hours during mitotic arrest, eventually triggering MOMP [6], [15]–[17]. This is analogous to the signaling through slow accumulation of active Caspase-8 that occurs during the extrinsic apoptosis pathway [18]. Based on this understanding, Gascoigne and Taylor proposed a pathway competition model to explain how cancer cells vary in fate following mitotic arrest. They hypothesized that cyclin B1 degradation and accumulation of the pro-death signal occur simultaneously, and that their outcomes are mutually exclusive. Cells that slip out of mitosis cease producing the pro-death signal and survive the mitotic arrest, whereas cells that initiate MOMP before they exit mitosis are committed to die [7]. Whether a cell slips and survives or dies in mitosis is determined by the relative rates of the two pathways, which can vary in individual cells.

An important question yet to be resolved is whether the two pathways are independent or mechanistically coupled when they compete for cell fates during prolonged mitosis (Figure 1B). Gascoigne and Taylor proposed the independent pathway model by showing that the fraction of cells that slip or die can be altered by perturbing the kinetics of either pathway [7]. The fact that we were able to trigger cell death by blocking slippage with Cdc20 siRNA in our previous study also suggests that slippage and death pathways are mechanistically independent to a certain degree [9]. However, these studies did not rule out the possibility that there may be crosstalk between the signaling pathways. Previous reports in the literature suggest at least two possible ways that the two pathways could be cross-linked. Kim et al proposed that the death pathway directly promotes slippage, by caspase-dependent proteolysis of the SAC protein BubR1 [19]. Allan and Clark showed that Caspase 9 is phosphorylated and inactivated by Cdk1/cyclin B1 in mitosis [20]. They thus proposed that gradual degradation of cyclin B1 resulted in gradual activation of caspase 9. Such crosstalk between the pathways may not be strong enough to completely activate/inactivate the pathway, but it may very well accelerate/delay its kinetics. In this study, we sought to investigate the kinetic interdependence of the two pathways and quantitatively distinguish between the independent and coupled pathway models by single-cell time-lapse experiments and simulations.

To investigate interdependence of the death and slippage pathways, we chose four cancer-derived cell lines from a larger panel we previously profiled [6] to span the full range of death-sensitivity in response to anti-mitotic drugs, including HeLa, MDA-MB-435S, A549 and MCF7 (listed in descending order of death sensitivity). We used a Kinesin-5 inhibitor (K5I), EMD534085, to induce mitotic arrest, as the action of this drug is specific to mitosis, unlike paclitaxel which also perturbs interphase microtubules. Kinesin-5 inhibitors promote cell death mainly during mitotic arrest, whereas paclitaxel promotes also high level of death after slippage [6], [9]. This may make paclitaxel more clinically active, but it would complicate our study, where we wish to focus on decision-making during mitotic arrest exclusively. We have seen very similar responses to EMD534085 and s-trityl-cysteine, a commercially available Kinesin-5 inhibitor [8].

## Results

### Kinetics of mitotic slippage in the absence of cell death

**Figure 2:**
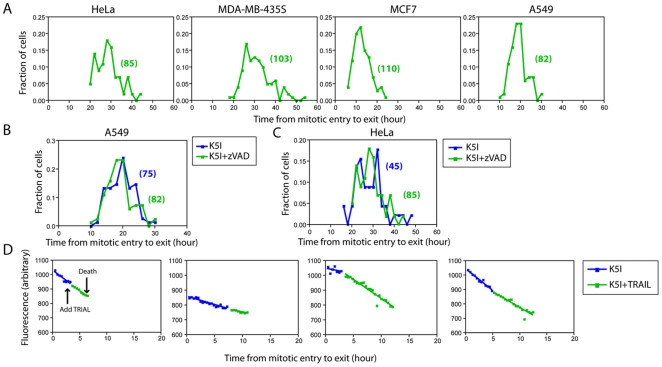
Slippage kinetics under perturbation of cell death. (A) Probability distributions of time duration from mitotic entry to exit for four cell lines treated with Kinesin-5 inhibitor (K5I) and zVAD-fmk. Numbers in parentheses indicate number of cells scored. (B) Probability distributions of time duration from mitotic entry to exit in A549 cells treated with K5I alone (blue) or K5I plus zVAD-fmk (green). Numbers in parentheses indicate number of cells scored. (C) Probability distributions of time duration from mitotic entry to exit in HeLa cells treated with K5I alone (blue) or K5I plus zVAD-fmk (green). Only cells that exited mitosis were scored for both treatments (for K5I alone: less than 10%; with zVAD-fmk: more than 90%). Numbers in parentheses indicate number of cells scored. (D) Four representative cyclin B1 degradation curves before and after the addition of TRAIL in HeLa cells treated with K5I. Average of whole-cell fluorescent intensity was measured using Region Statistics function in MetaMorph. Degradation rates before and after TRAIL addition were calculated using Linear Regression function in GraphPad Prism 4.

To measure kinetics of mitotic slippage without the presence of cell death, we used a broad spectrum caspase inhibitor, zVAD-fmk, to block caspase activity. This treatment blocked cell death in >90% of cells in all lines over the duration of our experiments, which allowed us to test pathway interdependence. Since this drug does not block MOMP, cells might die eventually, but using different pathways that we do not consider in our model. We scored the time interval between mitotic entry and slippage in unsynchronized populations using time-lapse phase-contrast imaging as described [8], [9]. Figure S1 showed representative time-series images of HeLa and A549 cells treated with K5I. From the single-cell movies, probability distributions of duration of mitotic arrest were quantified and results were shown for the four chosen cell lines in Figure 2A. In all cases, 100% of cells scored eventually slipped out of mitosis. Average arrest durations were: HeLa 28.4 hr, MDA-MB-435S 30.4 hr, MCF7 12.4 hr, A549 18.6 hr. The shape of the distributions appeared approximately normal in each case, with the shortest duration of mitotic exit much larger than zero. This distribution is consistent with the previously proposed ramp-threshold model for slippage kinetics [5]: at the time of mitotic entry, level of cyclin B1 starts to decrease (i.e. ramp down) with approximately linear kinetics, until it drops below a threshold that is required to sustain mitotic arrest [7], [9]. Variance in the observed population distribution of arrest time was probably resulted from variation in cyclin B1 degradation rate, or starting concentration, in individual cells.

Less than 10% A549 cells undergo cell death during mitotic arrest in K5I alone, so we could directly compare the duration of arrest with and without zVAD-fmk to test for pathway dependence. The distributions of arrest duration were very similar (Fig. 2B) and a statistical test failed to show a significant difference between them (p = 0.42). This observation strongly negates models in which slippage depends on caspase activation [19], and is consistent with the independent pathway model. A simple comparison of slippage kinetics +/− zVAD-fmk cannot be made across the whole population in more death-sensitive lines because many cells die during mitotic arrest in K5I alone. However, we can compare slippage kinetics for the fraction of cells that do not die in drug alone. Figure 2C showed this comparison for HeLa cells, of which <10% of cells survive mitotic arrest in K5I alone. Comparison of this minority population in K5I alone with the whole population in K5I + zVAD-fmk showed very similar distributions of mitotic arrest duration (p = 0.43), again arguing for pathway independence.

### Cyclin B1 degradation kinetics are insensitive to perturbation of the death pathway

Another way to test for pathway interdependence is to accelerate one pathway, and look for effects on kinetics of the other. This is possible for the cell death pathway by adding an additional pro-death signal that converges with the pro-death pathway activated by mitotic arrest. We added TRAIL (tumor necrosis factor (TNF)-related apoptosis-inducing ligand) as the additional pro-death signal. TRAIL activates the extrinsic death pathway, which converges on the intrinsic pathway at the level of triggering MOMP and/or by directly activating Caspase 3 [21]. We empirically determined a TRAIL concentration sufficient to speed up cell death in mitosis, but insufficient to trigger rapid death in interphase. Because cells died faster in mitosis when TRAIL was added, we could not use mitotic exit to monitor the slippage pathway. Instead, we measured cyclin B1 degradation directly, by infecting HeLa cells with adenovirus expressing full-length cyclin B1 fused to EGFP [22]. We chose to express this exogenous cyclin B1 reporter at a level close to the endogenous cyclin B1 (Fig. S2A, 1∶100) so the amount of total cyclin B1 was roughly 2-fold of the endogenous level in all our experiments. Cyclin B1 levels were monitored in real time by time-lapse fluorescence microscopy. K5I was added at the start of the imaging experiment. After the majority of cells had entered mitosis (judged by cell rounding), TRAIL was added to accelerate caspase activation and cell death. The average time from mitotic entry to death was 11.6 hr when TRAIL was added, significantly shorter than the death time of 18.0 hr for K5I treatment alone.

Figure 2D showed four representative single-cell fluorescence trajectories of EGFP-cyclin B1. Fluorescence of this cyclin B1 reporter, which is proportional to its concentration, decreased roughly linearly in time in mitotic arrest as previously reported [9]. After TRAIL addition, cyclin B1 continued to decrease linearly, with a rate that appeared to be not significantly different from that without TRAIL. In order to quantify the rate change of cyclin B1 degradation instead of relying on visual inspection of the traces, we calculated the normalized rate change for every individual cell, defined as (|k_b_|-|k_a_|)/|k_a_|, where k_a_ and k_b_ are cyclin B1 degradation rates before and after TRAIL addition (note that we assumed linear, not exponential, degradation kinetics in estimating these rates from the data). For the 27 cells that we analyzed, cyclin B1 degradation may be both slightly accelerated (12 out 27) and decelerated (15 out of 27) after TRAIL addition. Population average of the normalized rate change was calculated to be 0.11±0.59, suggesting the rate of cyclin B1 degradation on average increased by 11% after TRAIL addition. We considered such change was relatively small. Together with the observation that TRAIL addition may both accelerate and decelerate cyclin B1 degradation in individual cells, i.e. it triggers no universal trend, we conclude that addition of TRAIL did not alter kinetics of cyclin degradation, or mitotic exit.

We also measured, at the ensemble level, degradation kinetics of endogenous cyclin B1 in mitotic arrest with and without zVAD-fmk using western blotting. To conduct the experiment in mitotic-arrest cells, we treated HeLa cells with K5I for 3 hours, gently shook off the mitotic fraction of cells, then continued to culture cells in K5I or K5I + zVAD-fmk. Time series of cell lysates were obtained to perform immunoblots. As shown in figure S2B, along with quantification of the blot in figure S2C, inhibition of cell death in mitosis by zVAD-fmk did not alter the degradation kinetics of endogenous cyclin B1, further supporting the model that these two pathways are independent. In summary, our data all point to pathway independence, in which perturbation of the death pathway by either inhibition with zVAD-fmk or super-activation with TRAIL did not affect kinetics of mitotic slippage.

### Kinetics of cell death in the absence of mitotic slippage

**Figure 3:**
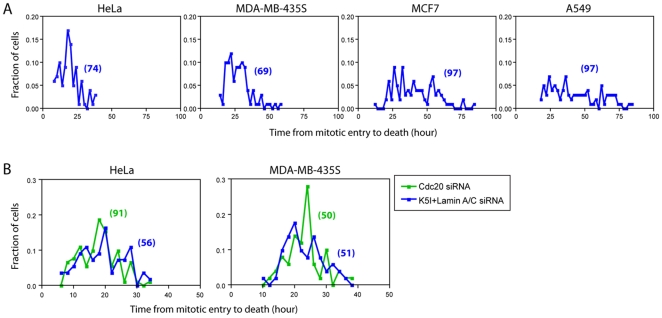
Death kinetics under perturbation of mitotic slippage. (A) Probability distributions of time duration from mitotic entry to death for four cell lines treated with Cdc20 siRNA and K5I. Numbers in parentheses indicate number of cells scored. (B) Probability distributions of time duration from mitotic entry to death under K5I plus Lamin A/C siRNA (blue) or Cdc20 siRNA (green). Only cells that died in mitosis were scored for K5I treatment (for HeLa: 90.3%, for MDA: 77.3%, (9)). Only the corresponding percentages of early mitotic death events were scored for Cdc20 siRNA treatment. Numbers in parentheses indicate number of cells scored.

To measure kinetics of cell death without the presence of mitotic slippage, we knocked down Cdc20 by siRNA treatment to block cyclin B1 proteolysis. We showed previously that this treatment is effective and specific, though it is important to control for some non-specific toxicity of the transfection protocol using control siRNA treatment [9]. We again scored the time interval between mitotic entry and cell death in unsynchronized populations using time-lapse phase-contrast imaging. Following Cdc20 siRNA treatment, 100% of cells died during mitotic arrest in all four cell lines and none slipped, indicating we had completely blocked the slippage pathway, and thus eliminated intra-cell line variation in fate choice. Figure 3A reported the population distributions of time between mitotic entry and death for the four lines. Average times for induction of death were: HeLa 18.0 hr, MDA-MB-435S 24.3 hr, MCF7 39.8 hr, A549 40.0 hr. The histograms were quite broad, reflecting high intra-cell line variation in death kinetics. The left side of each histogram had a large distance from zero, consistent with a ramp-threshold model for the kinetics of the pro-death pathway, similar to those of the slippage pathway, except that we do not know the identity of the molecule(s) that changes over time.

We reported previously that in the most death-sensitive line, HeLa, blocking slippage had little effect on death kinetics [9]. Here, we re-plotted the fraction of cells that died in mitosis under K5I, and the corresponding percentage of early mitotic death events under Cdc20 knockdown, as probability distributions, for HeLa and MDA-MB-435S. Death in mitosis is the dominant cell fate for these two death-sensitive cell lines, thus allowing quantification of sufficient dead cells for such statistical analysis. Distributions of time from mitotic arrest to death were similar under both K5I and Cdc20 RNAi for the two lines, as the calculated p values under the two conditions were not statistically significant (i.e. HeLa: p = 0.24; MDA-MB-435S: p = 0.78). This suggests loss of the slippage pathway did not affect death kinetics. The same kinetic comparisons for MCF-7 and A549 were re-plotted as survival curves in supplementary figure S3. It is evident that the initial drop in the curves, which corresponds to cell death in mitosis, showed similar kinetics under both K5I and Cdc20 RNAi. We chose to show the kinetics as survival curves instead of probability distributions for MCF7 and A549, as these two cell lines are death-resistant so death in mitosis is a rare event and we did not have enough data to generate the probability distribution curves. All the above experimental data showed kinetics of cell death were not perturbed by inhibition of mitotic slippage, again supporting a kinetic model of pathway independence.

### Simulation of single-cell statistics of mitotic cell fate

**Figure 4:**
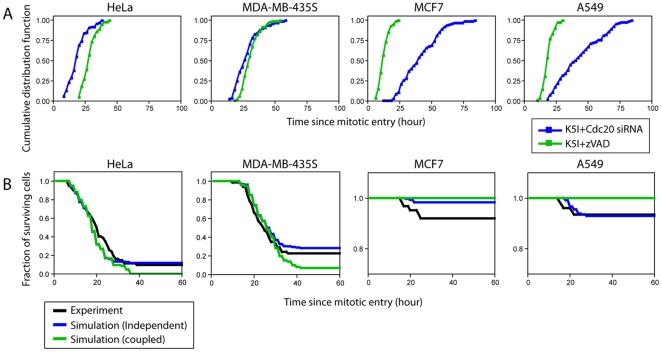
Simulations assuming independent or coupled pathway competition. (A) Cumulative distribution functions of mitotic death (blue) and slippage (green) for four cell lines from experimental measurement. (B) Cumulative survival curves for independent (blue) and coupled (green) pathway competition, averaged over ten sets of simulated data (each consist of 100 single-cell events). The corresponding experimental curve is shown in black.

One limitation of the pathway perturbation approaches in Figures 2 and 3 is that we can only directly compare kinetics of one pathway in the presence vs. in the absence of the other when that pathway dominates kinetically, i.e. for slippage in A549 and death in HeLa. In other cases, the kinetically dominant pathway precludes measurement of the alternative pathway, except in a small fraction of cells that choose that fate (e.g. slippage in HeLa shown in Fig. 2C). We therefore turned to a simple simulation strategy to test models of different kinetic interdependence of the two pathways when both are operational. For simulation, we first re-plotted the lifetime distributions when only one pathway was functional while the other was blocked (Fig. 2A and 3A) in the form of cumulative distribution functions, which showed the fraction of cells that had either died or slipped as a function of time after entering mitotic arrest (Fig. 4A). Visual inspection of these plots already reveals general trends: in death-sensitive cell lines (HeLa and MDA-MB-435S), death typically occurs before slippage; while in death-resistant cell lines (MCF7 and A549), the reverse is true.

To simulate a given cell's behavior quantitatively, we randomly sampled times of mitotic death and slippage from the two one-pathway cumulative distributions. We scored a cell as dying or slipping depending on which event occurred first, and the time of the event was determined by the shorter time. We chose to simulate two distinct scenarios: kinetic competition of independent or coupled pathways. For simulation of independent pathway competition, mitotic death and slippage events were randomly and independently generated from their respective cumulative distributions. 100 single-cell events were generated per simulation. For each of the four cell lines we studied, several simulated survival curves were superimposed on one run of experimental data under K5I alone, where both pathways are active (Fig. S4). In general, we saw good agreement between simulated (averaged over 10 sets of simulations) and experimental data, though the simulation slightly under-estimated the final percentage of cell death in MDA-MB-435S and MCF7 (Fig. 4B, blue lines). For simulation of kinetically correlated pathway competition, mitotic death and slippage events were chosen in a correlated fashion. For example, if mitotic death event was first randomly generated, the corresponding slippage event was then specifically chosen from the cumulative distribution at a time duration region similar to the mitotic event, and vice versa. As shown in the green lines of Figure 4, simulation results of the correlated pathway model significantly over-estimated death in HeLa and MDA-MB-435S and failed to predict any death events in MCF7 and A549. Therefore, a kinetic model of stochastic competition between independent pathways appeared to better explain our experimental observations.

## Discussion

In this paper, we used experimental perturbation and kinetic simulation to test whether the two cell fate-determining processes induced by mitotic arrest, i.e. mitotic death and mitotic slippage, occur by pathways that are, or are not, mechanistically coupled and/or kinetically correlated. Our data of slippage kinetics when death was blocked, and death kinetics when slippage was blocked (Fig. 2 and 3), argue against mechanistic coupling. A stronger and more quantitative test of the mechanistic independence is to ask if the kinetics of one pathway are altered by blocking or accelerating the other. We could make these direct comparisons only experimentally for death in the death-sensitive lines, and slippage in the death-resistant lines. Indeed, the kinetics of one pathway were unaffected by blocking the other in these cases (Fig. 2B and 3B). A direct comparison of kinetics with and without blocking the alternative pathway was more generally possible for the small subset of cells that undergo the less prevalent pathway when both are operational. Again, we observed no effect of blocking one pathway on the kinetics of the other, but this is a weaker test, since it includes only a subset of cells that might not be typical. In addition, we also observed that accelerating caspase activation had no effect on the kinetics of cyclin B1 proteolysis in single cells (Fig. 2D), further substantiating the independence of the slippage and death pathways.

A subtler test of pathway independence is to ask if two pathways are kinetically correlated in individual cells. Kinetic correlation is likely if pathways share upstream events, but could occur for other reasons, for example if the kinetics of both pathways depend on some common external variables, such as cell size, protein synthesis rate etc. We approached this question using kinetic simulation, asking how well the rates of each pathway alone, measured when the other is blocked, predict cell behavior when both pathways are active. We observed a good fit of simulated to experimental data assuming uncorrelated kinetics (Fig. 4B). A simulation of correlated kinetics fit the data less well in all the cell lines, though the distinction is not sufficiently large to completely rule out some degree of kinetic correlation. Overall, our observations strongly favor the independent pathway model in Figure 1B. We conclude that the large inter- and intra-cell line variability in response to anti-mitotic drugs is due to stochastic competition between two mechanistically and kinetically independent pathways, both of whose kinetics can be approximated by a gradual change in some parameter until a threshold is crossed, subsequently triggering an irreversible change that blocks the other pathway. In the exit pathway the gradual change is in Cyclin B concentration, in the death pathway its biochemistry is not yet known.

An interesting consequence of independent pathway competition is that fairly small changes in kinetics of either pathway can cause large changes in average cell fate. Average time for execution of the slippage and death pathways both varied ∼2.5 fold across the 4 cell lines that we studied. However, this relatively small rate difference is enough that in a cell line where death is faster than slippage, >90% of cells die during mitotic arrest (HeLa), while in a cell line where slippage is faster than death, <10% of cells die (MCF7 and A549). Large variation in average cell fate despite relatively small variation in pathway may explain why the cell death response to anti-mitotic drugs varies strongly across cancer cell lines [6], and why individual cells within a line can exhibit different fates. It may contribute to response variation in anti-mitotics seen in the clinic [23], and might also help explain why clinical resistance seems to arise rapidly to current anti-cancer drugs, including anti-mitotics.

So what is the mechanistic source of the rate variation we observe? We know from experimental measurements that most proteins vary up to 2-fold in concentration between individual cells in clonal populations of human cancer cells in culture, presumably due to stochasticity in gene expression and protein degradation [24]. Spencer et al argued that this kind of variation is sufficient to explain intra-line variation in response to TRAIL [25]. In the case that we study, noise in protein degradation and protein phosphorylation are likely to be key factors in causing cell-to-cell variation, as mitosis is a hyper-phosphorylated state where transcription is silenced and translation significantly attenuated. Protein degradation rate is known to control slippage kinetics [10]. It might also control the kinetics of apoptosis activation, though since our data favors uncorrelated kinetics for the two pathways, it would likely be proteolysis regulated in a different way. Cell-to-cell variation in proteolysis rates is an important topic for future research.

Many studies have revealed variation in sensitivity to apoptosis between cancer cell lines and investigated their origins [26], and it is easy to imagine that cancer clones are selected for decreased apoptosis sensitivity. Perhaps surprisingly, we observed just as much inter-cell line variation in slippage rates as we did in apoptosis rates (∼2.5 fold in both cases), though we know of no reason drug-naïve cancer cells should be selected for altered slippage rates. Could this degree of variation in average rate between clonal cell lines be typical for complex cellular pathways? Our study, along with other recent work [25], shows that intra-line variation in pathway rates can cause significant variation in cell fate in response to drug, which presumably contributes to the difficulty of completely eradicating cancer using drug treatments.

## Materials and Methods

### Cell culture and virus

All cell lines were purchased from American Type Culture Collection (ATCC, USA) and cultured under 37°C and 5% CO_2_ in appropriate medium supplemented with 10% fetal calf serum (FCS), 100 U/ml penicillin and 100 µg/ml streptomycin. HeLa and MDA-MB-435S were maintained in DMEM; MCF7 was maintained in RPMI; A549 was maintained in F-12K. Adenovirus expressing full-length cyclin-B1-EGFP was a gift from Randy King (Harvard Medical School).

### Chemicals and siRNA

Pan-caspase inhibitor, zVAD-fmk was purchased from Calbiochem and used at 100 µM. A potent and selective Kinesin-5 inhibitor (EMD534085) was provided by Merck-Serono. Super*Killer* TRAIL was a gift from Peter Sorger (Harvard Medical School) and used at 0.1 µl/ml. To deplete Cdc20, Ambion Silencer Select siRNA against Cdc20 (s2748) was used in all experiments at a final concentration of 50–100 nM. Dharmacon Lamin A/C siRNA duplex was used as control. siRNA transfection was performed using HiPerFect (Qiagen) according to manufacturer's instructions.

### Immunoblot Analysis

Cell lysates obtained using LDS sample buffer (NuPAGE, Invitrogen) were resolved on 10% Tris-HEPES gels (Pierce) and transferred to nitrocellulose membranes. Immunoblotting was performed according to manufacturers' recommendations using enhanced chemiluminescence (Amersham). Antibodies against cyclin B1 were purchased from BD Bioscience; PARP from Cell Signaling; tubulin from Sigma.

### Time-lapse imaging

Cells were seeded in glass-bottom plates (MatTek) in CO_2_-independent medium (Invitrogen) supplemented with 10% FBS, 100 U/ml penicillin and 100 µg/ml streptomycin. For fluorescent time-lapse imaging cells were seeded in phenol red-free CO_2_-independent medium (Invitrogen). Image acquisition was performed using Nikon TE2000 automated inverted microscope with a 20× objective enclosed in a humidified incubation chamber maintained at 37°C. Images were collected every 15–30 min using a motorized stage. Images were viewed and analyzed using MetaMorph software (Molecular Devices).

### Simulation of cell fate in mitosis

For simulation of independent pathways competition, two independent random numbers (uniformly distributed between 0 and 1), one corresponding to death (R_D_) and the other corresponding to slippage (R_S_), were generated using the Random function in MATLAB. R_D_ and R_S_ were used to sample time from mitotic entry to mitotic death (T_D_) and time from mitotic entry to mitotic slippage (T_S_), respectively. The probability of observing a mitotic death at time T_D_ was given by R_D_ *=  f_D_(*T_D_ *)*, which was the cumulative lifetime distribution function (discrete function with bin size  = 2 hr) obtained from our experiments (Fig. 4A, blue lines). Similarly, the probability of observing a mitotic slippage at time T_S_ was given by R_S_ *=  f_S_(*T_S_ *)* (Fig. 4A, green lines). Now we had two randomly generated T_D_ and T_S_ sampled from the distribution functions, cell fate was then determined by the relative value of T_D_ and T_S_. If T_D_ < T_S_, meaning death occurs before slippage, then this simulated event was scored as “death”. On the other hand, if T_S_ < T_D_, meaning slippage happens before death, this simulated event was scored as “slippage”. For simulation of coupled pathways competition, one random number (R) was generated to sample both T_D_ and T_S_. The probability of observing a mitotic death at time T_D_ and mitotic slippage at time T_S_ were given by R * =  f_D_(*T_D_ *)* and R * =  f_S_(*T_S_ *)*, respectively. Cell fate is again determined by the relative value of T_D_ and T_S_.

## Supporting Information

Figure S1 Time sequence of phase-contrast images of HeLa and A549 cells under K5I treatment. Numbers show elapsed time (hour:minute) relative to mitotic entry.
(TIF)

Figure S2 Analysis of cyclin B1 level by immunoblotting. (A) Comparison of endogenous (60 KDa) and exogenous (87 KDa) cyclin B1 level. HeLa cells were infected by adenovirus expressing full-length cyclin-B1-EGFP with indicated adenovirus-to-media ratio. Cell lysates were collected for immunoblots of cyclin B1 and tubulin (loading control). (B) Cyclin B1 degradation kinetics under K5I or K5I plus zVAD-fmk analyzed by immunoblotting. HeLa cells were treated with K5I for 3 hours, and mitotic fraction were shaken off and continued to be cultured in K5I or K5I plus zVAD-fmk with indicated times. Time series lysates were collected for immunoblot of PARP, cyclin B1 and tubulin. (C) Quantification of cyclin B1 degradation kinetics from (B). Area and mean intensity of inverted cyclin B1 bands were measured by ImageJ, and cyclin B1 level was defined as area multiplied by mean intensity. Cyclin B1 levels were normalized by the initial level (3 hours) and plotted as a function of time since mitotic entry.
(TIF)

Figure S3 Cumulative survival curves for indicated treatments in MCF7 and A549 (data from [9]).
(TIF)

Figure S4 Representative cumulative survival curves for simulated independent pathway competition. Each line consists of 100 single-cell events. The corresponding experimental curves are shown in black.
(TIF)
