# Supplementary figures and images for: Stochastic Competition between Mechanistically Independent Slippage and Death Pathways Determines Cell Fate during Mitotic Arrest
Source: PLoS One. 2010 Dec 21;5(12):e15724. doi: 10.1371/journal.pone.0015724 (PMC3006339; doi:10.1371/journal.pone.0015724)

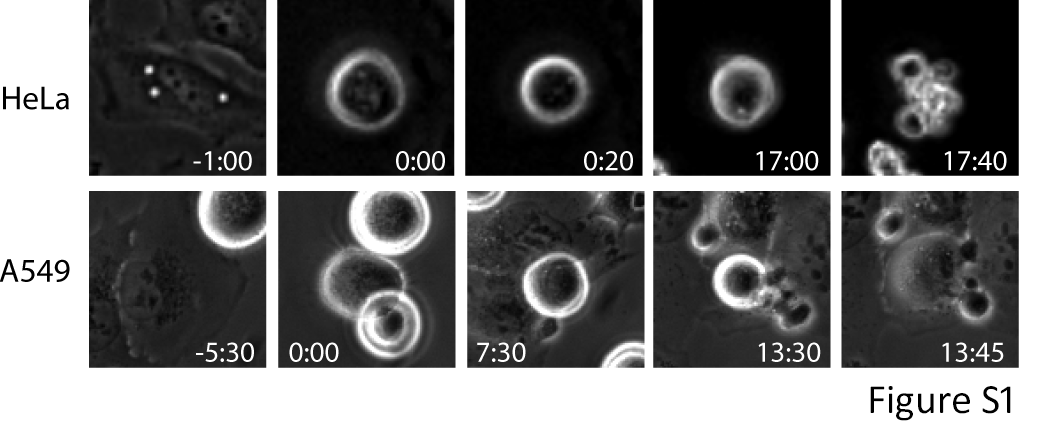

Supplement: Figure S1 — Time sequence of phase-contrast images of HeLa and A549 cells under K5I treatment. Numbers show elapsed time (hour:minute) relative to mitotic entry. (TIF) [file pone.0015724.s001.tif]

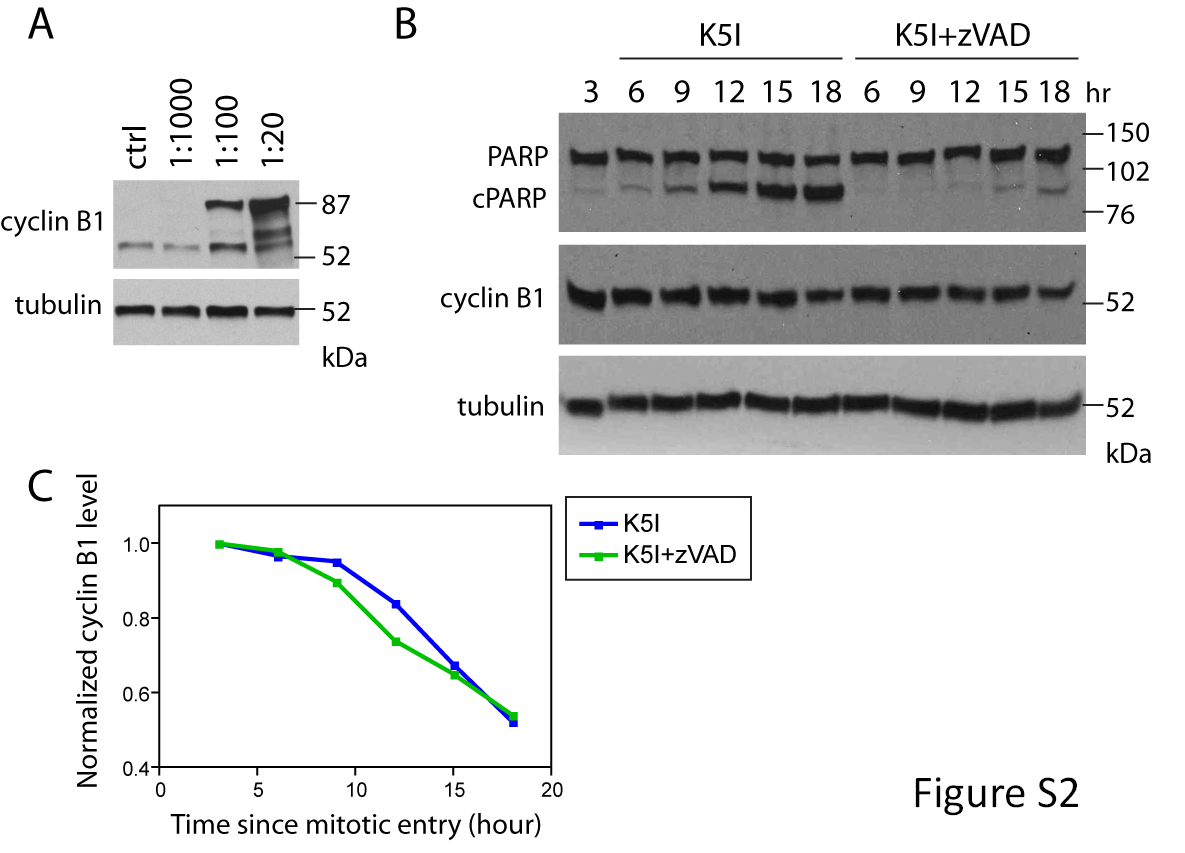

Supplement: Figure S2 — Analysis of cyclin B1 level by immunoblotting. (A) Comparison of endogenous (60 KDa) and exogenous (87 KDa) cyclin B1 level. HeLa cells were infected by adenovirus expressing full-length cyclin-B1-EGFP with indicated adenovirus-to-media ratio. Cell lysates were collected for immunoblots of cyclin B1 and tubulin (loading control). (B) Cyclin B1 degradation kinetics under K5I or K5I plus zVAD-fmk analyzed by immunoblotting. HeLa cells were treated with K5I for 3 hours, and mitotic fraction were shaken off and continued to be cultured in K5I or K5I plus zVAD-fmk with indicated times. Time series lysates were collected for immunoblot of PARP, cyclin B1 and tubulin. (C) Quantification of cyclin B1 degradation kinetics from (B). Area and mean intensity of inverted cyclin B1 bands were measured by ImageJ, and cyclin B1 level was defined as area multiplied by mean intensity. Cyclin B1 levels were normalized by the initial level (3 hours) and plotted as a function of time since mitotic entry. (TIF) [file pone.0015724.s002.tif]

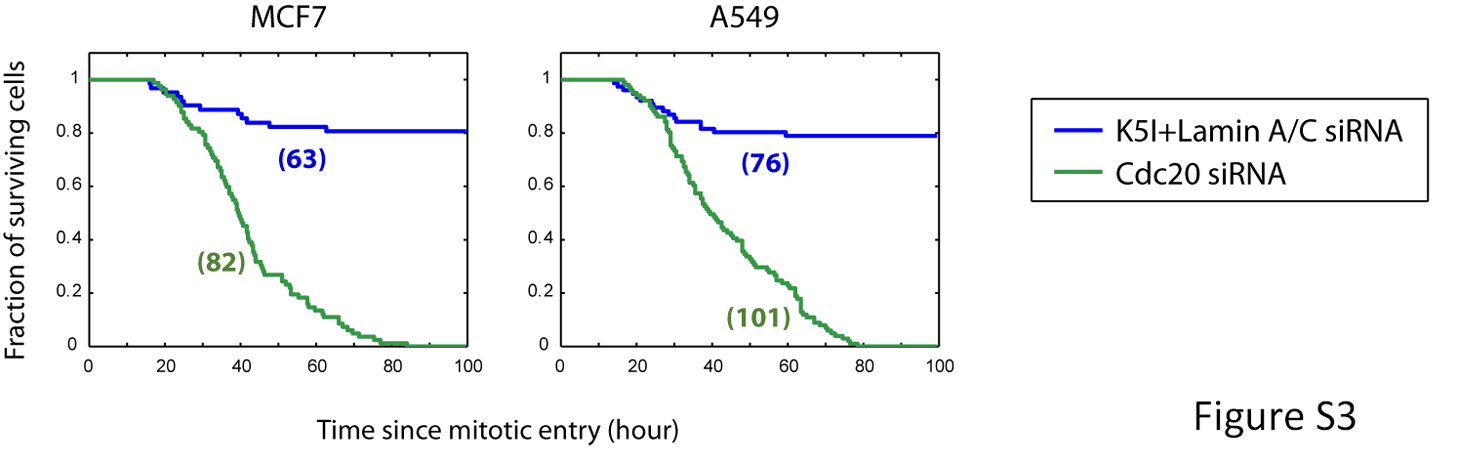

Supplement: Figure S3 — Cumulative survival curves for indicated treatments in MCF7 and A549 (data from [9]). (TIF) [file pone.0015724.s003.tif]

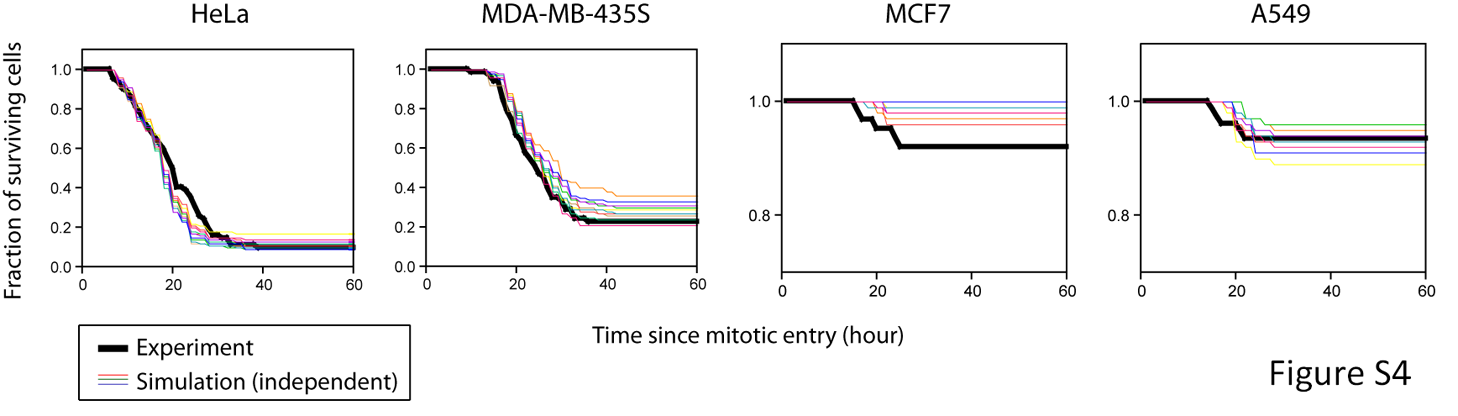

Supplement: Figure S4 — Representative cumulative survival curves for simulated independent pathway competition. Each line consists of 100 single-cell events. The corresponding experimental curves are shown in black. (TIF) [file pone.0015724.s004.tif]
